# Bone Mineral Density, Bone Biomarkers, and Joints in Acute, Post, and Long COVID-19: A Systematic Review

**DOI:** 10.3390/v16111694

**Published:** 2024-10-30

**Authors:** Fahad Alghamdi, Kinan Mokbel, Robert Meertens, Abasiama Dick Obotiba, Mansour Alharbi, Karen M. Knapp, William David Strain

**Affiliations:** 1 College of Medicine and Health, University of Exeter, Exeter EX2 4TH, UK; k.a.mokbel@exeter.ac.uk (K.M.); r.m.meertens@exeter.ac.uk (R.M.); a.obotiba@exeter.ac.uk (A.D.O.); k.m.knapp@exeter.ac.uk (K.M.K.); d.strain@exeter.ac.uk (W.D.S.); 2 Department of Radiologic Technology, College of Applied Medical Sciences, Qassim University, Buraydah 52571, Saudi Arabia; 3 PACS Admin, Radiology Department, King Khalid Hospital in Kharij, Riyadh 11942, Saudi Arabia; mansour_004@hotmail.com

**Keywords:** COVID-19, Post-COVID, long COVID, bone biomarker, DXA, bone mineral density, joints, MSK ultrasound

## Abstract

SARS-CoV-2 is highly transmissible and affects the respiratory system. People with COVID-19 are at higher risk of physical and mental health conditions, which could impact bone health. The aim of this review was to explore the effects of COVID-19 on BMD, BTMs, and joints. An electronic search of the PubMed, Web of Science, Scopus, and Ovid Medline databases considered studies published between 1 January 2020 and 1 November 2023. The search was limited to English, original studies in adult humans. The title and abstract of the identified papers were screened, followed by a full-text review using inclusion and exclusion criteria. The data extracted included the study and participant characteristics, BTMs, BMD, and joint abnormalities. The Newcastle–Ottawa scale quality assessment tool was used to assess the risk of bias. Five studies involving 305 out of 495 infected individuals observed a reduced BMD after COVID-19, with the most significant reduction occurring a year later. Both bone resorption and bone formation markers decreased, while regulatory markers showed higher levels in infected patients. COVID-19 may harm bone health by increasing bone regulatory markers and reducing bone formation and absorption, leading to a lower BMD. Elderly, frail, and osteopenic or osteoporotic individuals are at higher risk and should be regularly monitored for bone loss if they have long COVID.

## 1. Introduction

In early 2020, the world witnessed the outbreak of coronavirus disease (COVID-19), a highly infectious disease caused by the severe acute respiratory syndrome coronavirus-2 (SARS-CoV-2) virus. COVID-19 is known to cause severe respiratory illnesses, including pneumonia and acute respiratory distress syndrome (ARDS). It is a multi-organ disease that can affect people in various ways [1]. According to the World Health Organization (WHO), there are more than 770 million confirmed cases worldwide [2] and over 22 million in the United Kingdom [3]. During the acute phase of COVID-19, the symptoms, severity, and recovery times can vary significantly. While some patients may be asymptomatic, others may experience classic respiratory symptoms, fever, or a range of other manifestations [4,5]. In severe cases, hospitalisation, ventilation, and intensive care unit (ICU) admission may be required [6].

Throughout the COVID-19 crisis, some individuals who had recovered from the acute stages of the disease experienced unanticipated prolonged changes in their physical health and well-being [7]. The typical recovery time for COVID-19 ranges from 1 to 4 weeks. If symptoms persist for up to 12 weeks, it is classified as ongoing symptomatic COVID-19. If symptoms continue for more than 12 weeks without an alternative diagnosis, it is considered post-COVID-19 syndrome [8]. However, according to the National Institute for Health and Care Excellence (NICE), the term long COVID encompasses both ongoing symptomatic COVID-19 and post-COVID-19 syndrome [8].

COVID-19 presents a wide range of clinical features, with asymptomatic patients to those suffering from acute respiratory distress syndrome (ARDS) and multiple organ dysfunction [9,10]. Neurological, pulmonary, kidney, vascular, and cardiac manifestations have been extensively documented, but musculoskeletal involvement has only recently received attention, particularly concerning bones and joints. It is crucial to recognise that various respiratory conditions can adversely affect bone health, potentially leading to comorbidities such as bone loss and increased fragility.

Ageing leads to increased bone loss, resulting in complications such as osteoporosis, which significantly impacts a patient’s health [11]. The likelihood of osteoporotic fractures is approximately 10%, with a greater reduction in life expectancy compared to rheumatoid arthritis and some types of cancer [12,13]. Clinical reports suggest that patients with respiratory diseases may face a higher risk of fractures, although glucocorticoid use is frequently a key contributing factor in this group [14,15]. Many disorders can cause secondary bone loss and fragility. Osteoporosis is a chronic condition characterised by a progressive loss of bone density and microarchitectural deterioration, leading to an increased risk of fractures [16]. Various risk factors for osteoporosis and bone loss include gender, lifestyle, advanced age, steroid therapies, a low body mass index (BMI), hormonal disorders, chronic conditions, critical illness, and decreased mobility [17,18]. There are indications that SARS-CoV-2 may negatively affect bone health [19]. COVID-19 patients often have risk factors for bone fragility which include systemic inflammation, older age, glucocorticoid treatment, and mobility issues. Vertebral fractures (VFs) are associated with more severe respiratory function impairment compared to those without VFs [20]. The 30-day mortality risk increases from 12.8% without VFs to 20.7% with one VF and 34.8% with multiple VFs [21].

Joint pain can originate from a diverse array of causes, including both traumatic injuries and chronic conditions such as arthritis. Notably, respiratory infections have the potential to trigger either new-onset or exacerbations of rheumatoid arthritis [22]. Throughout the COVID-19 pandemic, instances of pain and stiffness were frequently reported, with arthralgia appearing alongside other symptoms related to the virus [23]. It has been observed that arthralgia may serve as an early indicator of COVID-19, affecting 10–15% of individuals diagnosed with the virus [24,25]. An increasing number of studies have documented the onset of various rheumatic musculoskeletal diseases that seem to develop in close temporal relation to COVID-19 infection. These conditions include rheumatoid arthritis, polymyalgia rheumatic, and reactive arthritis, among others [26,27,28]. One study indicated a significant prevalence of rheumatic and musculoskeletal symptoms among COVID-19 survivors following hospital discharge. In a study involving 285 participants, 74.6% reported at least one symptom three months post-discharge, with 39.2% specifically experiencing joint pain. By six months, the prevalence decreased to 43.2%, with 18.6% reporting joint pain [29]. In contrast, fewer than 10% of the individuals with post-COVID syndrome have been reported to exhibit these rheumatic symptoms [30].

Due to the limited understanding of the impact of COVID-19 on bone and joint health, the heterogeneous nature of this condition, and its association with the musculoskeletal system, the aim of this systematic review was to identify the potential negative effects of COVID-19 on bone and joint health and any existing gaps in the literature. This can help in identifying issues early in high-risk groups to prevent further complications and raise awareness. No ongoing systematic studies or scoping reviews on this subject were found following a preliminary search of PubMed, Web of Science, Scopus, and Medline Ovid.

## 2. Methods

### 2.1. Protocol

The PICO framework (Participant or Patient, Intervention, Comparison, and Outcome) was used to establish eligibility criteria. [31]. To report the literature in this systematic review, we utilised the Preferred Reporting Items for Systematic Reviews and Meta-Analyses (PRISMA) extension [32]. This systematic review was registered on the National Institute for Health and Care Research/International Prospective Register of Systematic Reviews PROSPERO Registries (https://www.crd.york.ac.uk/prospero/, accessed on 24 October 2024) (registration number: CRD42024540315).

### 2.2. Eligibility Criteria

#### 2.2.1. Inclusion Criteria

This systematic review included observational studies involving individuals of any sex and ethnicity across the three stages of COVID-19: acute, post-acute, and long COVID-19. Studies with or without healthy control groups were eligible, with study designs including cross-sectional, case–control, prospective, and retrospective cohort studies. Eligible studies were published between 1 January 2020, and 1 November 2023, with no geographic restrictions, provided that the abstract or full-text article was available in English. The review included studies that examined at least one bone turnover marker (BTM) and/or bone mineral density (BMD), where BMD was assessed using dual-energy X-ray absorptiometry (DXA) or computed tomography (CT). Only studies reporting BMD in T-scores or mean values, using either DXA or CT, were included. Due to the anticipated limited number of CT studies assessing the impact of COVID-19 on BMD, we relaxed our criteria to include all the studies reporting T-scores or mean values. For joint health, only studies that assessed joints using diagnostic medical ultrasound were included. For studies on acute COVID-19, only those that included follow-up assessments were considered.

#### 2.2.2. Exclusion Criteria

This review did not consider descriptive observational study designs, such as case series, individual case reports, or descriptive cross-sectional studies. Non-original studies, including reviews, editorials, commentaries, book chapters, conference proceedings, and papers without relevant data, were also excluded. Studies with imprecise results or data that could not be extracted were excluded as well. Additionally, studies that unreasonably attributed long COVID diagnoses to the sick group of the study population were excluded. Furthermore, studies involving musculoskeletal ultrasounds for tissues other than joints (e.g., muscle, diaphragm, etc.), clinical pharmacological intervention trials, vaccinations that may impact data interpretation, and studies using DXA scanners to measure total body composition or BMD via quantitative ultrasound were excluded. We also excluded studies examining BMD in acute COVID-19 patients that did not assess these outcomes in follow-up measurements. Additionally, we likewise excluded studies investigating the effects of BMD, BTMs, and joint diseases on COVID-19. Studies with poor results due to technical issues were also excluded.

#### 2.2.3. Participants

The study population included those infected with COVID-19: acute or post-COVID syndrome or long COVID.

#### 2.2.4. Context

The studies assessed BTMs, BMD, and ultrasounds of joint abnormalities in the COVID-19 population.

#### 2.2.5. Types of Sources

To the best of our knowledge, no systematic reviews or meta-analyses have been conducted on the topic of this systematic review’s research question. Further information regarding the types of sources and the inclusion and exclusion criteria is covered in Section 2.2.1 and Section 2.2.2.

### 2.3. Information Sources

A comprehensive search was conducted using the electronic literature databases the National Institutes of Health (NIH), the National Library of Medicine PubMed (https://pubmed.ncbi.nlm.nih.gov/), Medline Ovid (https://ovidsp.dc1.ovid.com/), Web of Science (https://www.webofscience.com/wos/woscc/basic-search), and Scopus (https://www-scopus-com.uoelibrary.idm.oclc.org/). These databases have been accessed on 1 November 2023 to find relevant papers published between 1 January 2020 and 1 November 2023. The study was conducted according to the Preferred Reporting Items for Systematic Reviews and Meta-Analysis (PRISMA 2020) guidelines [33].

### 2.4. Research Strategy

The search strategy was established in the PubMed database and then adapted to the other databases. Keywords (Table 1), MeSH terms, and Boolean operators for synonyms were used to build a comprehensive search syntax. This review used a broad combination of keywords, as shown in Table 1, to reduce the possibility of excluding pertinent studies with the search strategy.

### 2.5. Studies Selection

The articles from all the databases were compiled into an Excel spreadsheet to manage the identified abstracts and articles from the database searches and ensure that the bibliography was accurate. The main reviewer then removed any duplicate articles and assessed the results using the inclusion and exclusion criteria. The screening process determined whether the references in the included studies’ reference lists met the eligibility requirements and were published by the specified deadline. During the title and abstract screening, the reviewer used “Yes”, “No”, and “Maybe” to categorise the articles. A second reviewer checked the screening results to minimise bias. Subsequently, a third reviewer evaluated the selected articles to confirm that the inclusion and exclusion criteria were correctly applied and that all eligible articles were chosen. All selected publications were double-checked and reviewed by the reviewers/co-authors with backgrounds in medicine, medical imaging, and related fields.

### 2.6. Data Extraction from Included Studies

The primary reviewer meticulously organised the data from the included studies into three tables. The first table contains detailed information, such as the aims, participant demographics (age, gender, and number), COVID-19 status, history of musculoskeletal conditions, type of assessment, hospitalisation status, and follow-up duration. The second table outlines the bone mineral density (BMD) results based on the extracted measures (means or T-scores), the modality used, and the body part that was measured. These results are categorised by COVID-19 status and follow-up intervals. The third table presents the BTMs (bone resorption, formation, and regulatory markers) in relation to the COVID-19 status.

### 2.7. Risk of Bias Assessment

The risk of bias for each study was assessed using the Newcastle–Ottawa scale for cohort studies and control studies [34] and Newcastle–Ottawa Scale adapted for cross-sectional studies [35]; an overall risk of bias score was assigned to each study (low, moderate, or high) by the main reviewer.

## 3. Results

**Figure 1:**
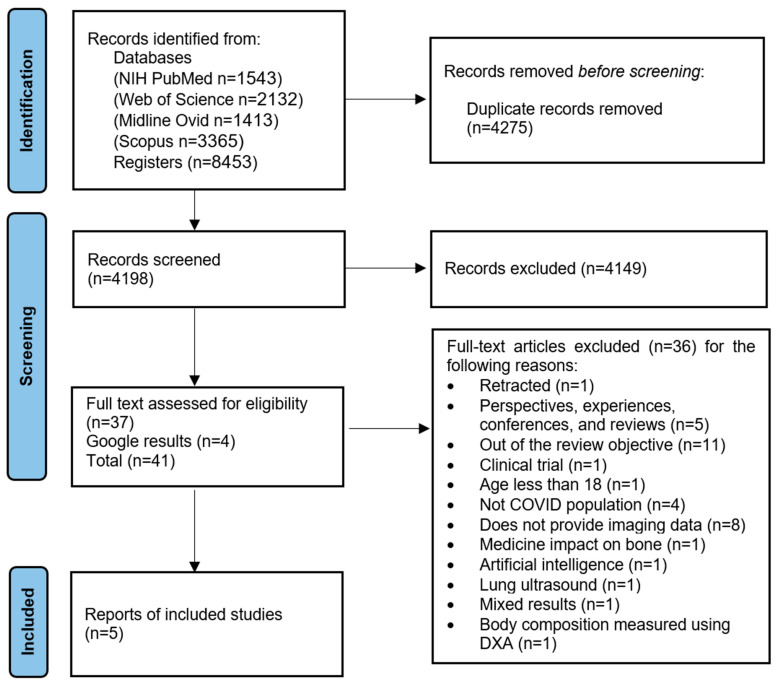
Flow chart showing the process of identifying eligible studies.

The research strategy yielded a total of 8453 studies that were retrieved across the four electronic databases (Figure 1): PubMed (1543), Web of Science (2132), Medline Ovid (1413), and Scopus (3365). An additional four records were identified during reference tracking of the selected papers using Google Scholar. A total of 4275 duplicate articles were excluded for having the same title/authors, resulting in 4198 references being screened based on their titles and abstracts. After this screening, 4149 records were excluded for not meeting the eligibility criteria. Subsequently, the full text was screened for 41 potentially eligible references. Finally, five eligible references that met the criteria were included in the systematic review.

### 3.1. Study Characteristics

All the included studies reported on SARS-CoV-2 on bone health and were published between 2020 and November 2023. The five studies included in this analysis evaluated bone health through BMD and BTMs. Unfortunately, no studies were eligible for inclusion that examined the impact of COVID-19 on joints using ultrasound. However, the results included one article focused on BTMs that are resorption, formation, and regulatory markers [36]; three focused on BMD [37,38,39]; and one had data on both BMD and a single regulator BTM [40]. Regarding the modality used for detecting BMD, three articles utilised DXA scene [37,39,40] and one used CT [38].

For the case group, three articles conducted their assessments on post-COVID-19 patients [37,39,40], one article on acute COVID-19 patients [36], and one article examined both acute and post-COVID-19 patients [38]. However, none of the studies explored the long COVID-19 population. For the comparative/control group, four articles compared their measures with a SARS-CoV-2-negative group [36,37,39,40] and one article utilised a repeated measures design [38]; overall, healthy participants took part in only two studies [36,40]. Furthermore, two of the studies involved patients with comorbidities in both groups. One study included patients with osteoporosis or osteopenia [37], and the other study involved patients with various musculoskeletal disorders (neurological or degenerative) [39]. Regarding hospitalisation, two studies examined hospitalised patients [36,39], and, in one study, some patients required intensive care during their baseline treatment [38]. Patients treated with steroids were excluded from two studies [38,40].

The timing of the BMD measurements varied across the studies. For instance, one study took three measurements within a span of three months after the COVID-19 infection as a baseline, followed by measurements at 9 months and 21 months after the baseline measurement [37]. Another study took measurements during hospitalisation with the initial CT and then again between 33 and 129 days later [38]. Moreover, one study measured BMD 3 months after recovery [40]. A separate study measured the BMD of COVID-19 patients one month after diagnosis [39]. The studies conducted included two single-centre retrospective studies [37,38], two case–control studies [36,40], and one prospective cross-sectional study [39]. The countries in which these studies were conducted were as follows: Austria [36], Saudi Arabia [37], Turkey [38], Romania [39], and Iraq [40]. Table 2 shows the characteristics of the included studies.

### 3.2. Impact of COVID-19 on Bone Mineral Density

The results showed that SARS-CoV-2 increased the likelihood of a negative impact on BMD results in the infected groups [37,38,39,40] [Table 3]. While CT was the primary modality identified for BMD measurement in studies screened during the systematic review, the majority of these CT-based studies were excluded because they did not specifically examine the effects of COVID-19 on BMD, thus not fulfilling our inclusion criteria [41,42,43,44,45,46,47,48].

The impact of COVID-19 on BMD was observed in hospitalised patients within the acute infection phase, and the BMD was found to be reduced by 8.6% (±10.5%). The proportion of patients with osteoporosis within the group doubled from 12.1% to 24.1% after an average follow-up of 81 (±48) days. In addition, the change in BMD was found to have a negative correlation with the length of hospital stay (r = −0.35, *p* = 0.010), with little difference observed between genders [38].

One month post-infection, patients hospitalised due to COVID-19 with various musculoskeletal disorders showed an osteoporotic rate of 98.83% compared to the control group [39]. After three months of infection, the osteoporotic and osteopenic populations receiving treatment reported reductions in BMD of −4.68% (−19.19 to 19.23) for the lumbar spine and −3.6% (−27.3 to 13.1) for the femur compared to baseline measurements. Notably, the patients most affected by COVID-19 exhibited the most pronounced BMD decreases compared to other patients with moderate and mild COVID-19 severity, with lumbar and femur reductions of −15% (−19.25 to −9.21) and −7.78% (−27.09 to −1.95), respectively [37].

Three months after recovering from COVID-19, individuals who had fully recovered showed a BMD reduction of −0.43 ± 0.94 compared to the control group [40].

Additionally, COVID-19 patients treated with corticosteroids showed a significant reduction (*p* = 0.008) in both lumbar and femur BMD [37], and patients who received a total corticosteroid dose of over 600 mg experienced a much greater reduction compared to those who were treated with a lower corticosteroid dose (*p* = 0.011). This reduction was associated with both the total corticosteroid dose (*p* = 0.014) and the duration of corticosteroid treatment (r = 0.27, *p* = 0.045) [38].

### 3.3. Impact of COVID-19 on Bone Turnover Markers

The levels of markers related to bone turnover, such as the C-terminal telopeptide of type 1 collagen (CTX-1) and osteocalcin (OC), were notably lower in COVID-19 patients than in the control group (0.172 [0.097; 0.375] vs. 0.462 [0.300; 0.649] for CTX-1, and 10.50 [6.49; 16.26] vs. 15.33 [11.85; 19.63] for OC, respectively) (Table 4). Nevertheless, the levels of sclerostin (SOST) and dickkopf-1 (Dkk1) were significantly higher in infected patients in comparison to the control (37.52 [25.30; 60.11] vs. 27.98 [24.05; 39.24] for SOST and 23.16 [19.77; 34.73] vs. 21.31 [15.04; 24.69] for Dkk1, respectively). Interestingly, there were no significant differences in the other BTMs between non-ICU SARS-CoV-2 hospitalised patients and the controls [36].

Furthermore, an observation indicated that the serum levels of osteoprotegerin (OPG) in post-COVID patients were elevated and had an inverse relationship with their BMD. It is worth mentioning that two potential studies, which were excluded, discussed some bone turnover markers; however, they were out of scope for this review [49,50,51].

### 3.4. Impact of COVID-19 on Joints

The screening results failed to identify any studies on the impact of SARS-CoV-2 on joints that met the eligibility criteria for this review. However, numerous case–control papers and case series are available, as well as a systematic review on related studies, including post-vaccine arthritis [52,53]. Two articles had the potential to be included in the review: one study combined ultrasound results from the study groups with isolated arthritis and “connective-like” arthritis [54], while the other did not include the sonographic presentation of joints [55].

### 3.5. Risk of Bias

Two researchers independently assessed the quality of cross-sectional and cohort studies using the Newcastle–Ottawa Scale (NOS), which provides a rating system using stars Any disagreements were resolved by a third author, and a consensus was achieved [Table 5].

## 4. Discussion

The primary goal of this systematic review was to systematically analyse the published data in the field and identify any existing gaps regarding the negative impact of COVID-19 on BMD, BTMs, and joint health. This was conducted to highlight the issues early on in high-risk groups and prevent further complications by raising awareness. In this systematic review, only five published studies were included; four provided data on BMD and two on BTMs, with 305 out of 495 people infected with SARS-CoV-2. The results from the observational studies revealed that individuals infected with SARS-CoV-2 had a decrease in BMD after infection [37,38,39,40], with the greatest reduction occurring after 9 months [37]. This can be explained by the imbalance of BTMs in the acute and post-acute phases [36,40], and the resultant changes in bone density, which take time to become apparent.

Two of these studies involved patients with comorbidities. One study focused on patients with osteopenia and osteoporosis, while the other included individuals with various neurological or degenerative musculoskeletal disorders. It is important to note that these conditions have a greater impact on bone health compared to healthy individuals, and this should be considered when comparing the outcomes of these two studies with those involving healthy participants [56].

An early prediction suggested that individuals who have contracted COVID-19, as well as those affected by safety measures or lockdowns, may experience negative effects on bone health [57]. Furthermore, some studies found that SARS-CoV-2 infection in non-human models has a detrimental impact on bone health [58,59,60]. Additionally, it has been suggested that the use of corticosteroids in COVID-19 patients may contribute to these physiological changes.

When comparing the BMD reductions between non-COVID corticosteroid patients and COVID corticosteroid patients, it was observed that the group with SARS-CoV-2 had a lower BMD [37]. Glucocorticoids are known to effectively treat acute respiratory distress syndrome (ARDS) by reducing inflammation and improving lung function [61]. They are commonly used for COVID-19 patients around the world [62]. However, these medications can accelerate bone loss, increasing the risk of osteoporosis. Therefore, it is crucial for clinicians and researchers to focus on the relationships between COVID-19, glucocorticoids, and osteoporosis, especially in elderly patients [63].

During the early stages of the pandemic, several studies aimed to establish a relationship between BMD and the necessity for intensive care, prognosis, and outcomes from chest CT scans. Some studies found no association [43,45,48]. However, one study discovered an inverse link between L1 BMD and the likelihood of death, which disappeared after adjusting for age [47]. Another study identified osteopenia in COVID-19 patients with moderate to severe pneumonia [44].

Bone density or BMD can be measured by several modalities: absorptiometry (single- or dual-energy, photon or x-ray) or quantitative CT [64,65]. These modalities are non-invasive but include radiation exposure and the precision and accuracy of these tests can vary from one modality to another. However, DXA scanners are considered the gold standard tool for gathering such information by scanning the lumbar spine (L1–L4) and hip region. It follows the World Health Organization’s osteoporosis T-score classification and the National Institute for Health and Care Excellence guidelines for fragility fracture risk assessment, can be used to observe healing responses, and has a reliable reference range [66,67,68].

It is known that changes in bone volume occur over time after the acute infection has resolved, while BTMs can change while the acute phase of the infection is still active. Assessing BTMs is a precise laboratory method that reflects specific changes in bone tissues during the different stages of bone remodelling in real time [69]. Bone resorption and formation are involved in bone remodelling; they are not separate but independently regulated processes, forming part of a specific temporary structure. Both processes involve bone marrow-derived precursors, which are regulate by growth factors and cytokines, and differentiated and developed by a number of systemic hormones as well as mechanical factors [70]. The balance between the amount of bone resorbed by osteoclasts and the amount produced by osteoblasts is crucial [71,72].

Monitoring bone markers is a highly sensitive way to track sudden changes in bone metabolism. It is valuable for evaluating treatment effectiveness, but it is not dependable for predicting outcomes on an individual basis [73]. When combined with BMD, bone markers are a good initial monitoring tool for groups of people rather than individuals since they only demonstrate weak correlations with BMD [74]. The International Osteoporosis Foundation (IOF) and the International Federation of Clinical Chemistry and Laboratory Medicine (IFCC) recommend one bone formation marker (serum procollagen type I N propeptide, s-PINP) and one bone resorption marker (serum C terminal cross-linking telopeptide of type I collagen, s-CTX) as reference markers. These should be measured by standardised assays in observational and intervention studies in order to enlarge the international experience of the application of markers to clinical medicine and to help resolve uncertainties over their clinical use [75].

A decreased level of circulating osteocalcin (OC) had been observed in critically ill ICU COVID-19 patients in comparison to non-COVID-19 patients [49]. Additionally, individuals with non-severe COVID-19 had lower levels of total procollagen type 1 amino-terminal propeptide (P1NP) and osteocalcin N-terminal in the middle (N-MID OC) compared to healthy individuals [50]. Another study found that the COVID-19 patient group had a lower level of serum OPG compared to the matched control group [60].

Cytokines produced by the immune system also play a crucial role in bone remodelling. These cytokines regulate the activity of osteoblasts, osteoclasts, and other bone cells and are involved in both bone formation and resorption; as the body ages, these inflammatory molecules are produced naturally. Ultimately, they are essential for maintaining the delicate balance of bone homeostasis [76]. In premenopausal women, there were notable but modest inverse correlations between interleukin (IL-6) and trochanter BMD, as well as C-reactive protein (CRP) and femoral neck and trochanter BMD. On the other hand, tumour necrosis factor (TNF-α) had a positive association with spine BMD in a cohort study [77]. Also, another study showed that there is a positive correlation between the production of IL-1, IL-6, and TNF-α by peripheral blood mononuclear cells and vertebral bone loss in healthy premenopausal women [78]. Furthermore, a study found that a group with decreased BMD exhibited a notably higher concentration of CRP. Similarly, the TNF-α concentration was also higher in the same group, although the difference was not statistically significant [79].

Our understanding of the pathophysiology of the impact of various factors on BMD is still in its early phase. Generally, one of the theories is that when the body contracts SARS-CoV-2, it triggers the production of cytokines (such as IL-17, TNF-α, and CXCL10) which in turn could lead to a decrease in BMD by promoting the loss of bone cells (i.e., osteoclastogenesis) and reducing the growth and differentiation of bone-building cells (i.e., osteoblasts) [80]. Another theory is that the virus either decreases the expression of ACE2 or increases the levels of angiotensin II. This can lead to inflammation and stimulation of the body’s immune response, causing widespread inflammation in various organs, including the skeletal system [81].

Any interruption or abnormalities in the bone remodelling cycle will result in skeletal disorders with low- or high-bone-mass syndromes, which increase the possibility of fracture or metabolic diseases for which the skeletal system is responsible [82,83]. A study investigating how COVID-19 may affect bones and whether it could lead to a higher risk of osteoporosis in the future found that blocking certain genes involved in bone growth, such as collagen I, osteocalcin (OCN), and Runt-related transcription factor 2 (Runx2), with increased expression of a microRNA (miR-4485-3p) affects bone marrow mesenchymal stem cells (BMSCs) [84].

Maintaining bone health is crucial, and vitamins play a significant role. Vitamin D, in particular, plays multiple roles in bone well-being and immune system regulation. Vitamin D plays an important role in maintaining bone density and preventing bone loss. It aids in mineralisation and calcium absorption by accessing calcium through the intestine and reabsorbing it in the kidneys when required. Additionally, it regulates calcium and phosphate levels and metabolism, which are crucial for bone formation and mineralisation. By promoting calcium deposition and inhibiting bone resorption, it contributes to the formation of hydroxyapatite, the mineral component of bones [85,86]. However, insufficient levels of vitamin D can cause bone loss as a result of an increase in serum parathyroid hormone (PTH) which stimulates bone turnover due to the lack of calcium [87,88].

Moreover, vitamin D showcases immunomodulatory characteristics that assist in regulating the immune system. It plays a crucial role in adjusting both the adaptive and innate immune systems by influencing cytokines and cell signalling pathways [89,90]. Studies have linked its deficiency to dysregulated immune responses and heightened vulnerability to respiratory infections [91]. Vitamin D also regulates the synthesis of antimicrobial peptides, enhances neutrophil activity, and maintains the lungs’ protective barriers [90,92]. It has been noted that vitamin D plays a role in suppressing autoimmune responses and has anti-inflammatory properties that help reduce the production of pro-inflammatory cytokines and furthermore, it increases the anti-inflammatory response [93,94].

Multiple studies have demonstrated the protective effects and benefits of vitamin D in COVID-19. Research had found that patients with COVID-19 who were supplemented with vitamin D and maintained a serum 25(OH)D level above 12 ng/mL experienced significantly lower rates of RT-PCR positivity, fewer ICU admissions, and reduced mortality. These findings underscore a clear association between adequate vitamin D levels and a diminished risk of ICU hospitalisation [95,96,97].

The extended lockdowns associated with the COVID-19 pandemic resulted in prolonged indoor confinement for many individuals, significantly restricting physical activity and sunlight exposure. Furthermore, severe COVID-19 cases often necessitated extended bed rest. Similar to astronauts, at-risk individuals may have experienced bone mass loss, leading to disruptions in bone turnover and contributing to systemic osteoporosis [98]. The research indicates that decreased physical activity adversely affects both bone and muscle function. For instance, patients undergoing total knee replacements and those who have experienced strokes frequently exhibit diminished muscle mass and increased bone loss, resulting in prolonged recovery periods that can extend beyond one year [99,100,101,102,103,104].

Hypoxia can lead to bone metabolism disturbance by promoting the differentiation of osteoclasts and further inflammation [105,106]. COVID-19 can cause dyspnoea, which is associated with reduced oxygen levels [107]. Additionally, the components of the fibrinolytic system play a role in various stages of viral infection, resulting in a range of complications. These may include thrombosis and fibrosis in immune-privileged tissues, leading to persistent inflammation and micro-clots. These clots can block micro-capillaries, hindering oxygen exchange and ultimately causing tissue hypoxia [108,109].

Similar to COVID-19, patients with chronic obstructive pulmonary disease (COPD) have a higher chance of developing osteoporosis. If the osteoporosis is severe, the risk of fractures increases fourfold, and those with severe dyspnoea face a doubled risk [110]. Additionally, individuals hospitalised for influenza are at a greater risk of experiencing fractures and falls after being discharged compared to people of the same age and gender who were admitted for different reasons [111]. Other viruses have also shown negative implications for bone health; for example, studies have associated human immunodeficiency virus (HIV) and hepatitis B virus with reduced BMD in infected individuals [112,113]. During the previous SARS-CoV-1 epidemic, patients were reported to have bone abnormalities and osteonecrosis, along with a reduced bone density during their recovery [114,115].

Joints can be affected by viral infections, leading to conditions such as reactive arthritis or monoarthritis due to secondary inflammation [116,117]. This is similar to other viral infections that can cause acute arthralgia and arthritis, including hepatitis B or C viruses, Epstein–Barr virus, and human immunodeficiency virus, among others [117,118]. Although efforts have been made to explore the possibility of COVID-19 causing complications in the joints, the exact mechanisms underlying this manifestation remain under investigation.

Furthermore, a study found that nearly two in every five long COVID patients report widespread pain in their joints and muscles, likely induced by the acute phase of COVID-19 [29]. This is potentially caused by the “cytokine storm” and very likely induced by the acute phase of COVID-19 [119,120]. Regarding the location of joint pain, some reports indicated that among patients who reported at least one rheumatic or musculoskeletal symptom following COVID-19 infection, joint pain was generalised in 64.2% of participants. When assessing the localisation of symptoms, the most commonly impacted regions were the knee, foot–ankle joint, and shoulder [29]. Another study found that 7.6% of patients experienced joint pain, predominantly in the knee, but also in the elbow, ankle, wrist, and spinal joints [121]

Additionally, one study indicated that cases of reactive arthritis following COVID-19 have a higher prevalence in females. These cases typically exhibited oligoarticular and asymmetrical involvement, with an average of three affected joints and a frequent co-occurrence of axial symptoms and enthesitis. The knee was the most affected joint (69% of patients, 39% bilateral), followed by the ankle in 65% and the wrist in 30% of patients. Inflammatory lower back pain was reported in 30% of patients, while 39% exhibited enthesitis, primarily in the Achilles tendon and chest wall [55].

In addition, Mukarram et al. provided a detailed description of patients who developed symmetrical polyarthritis following COVID-19 infection. These patients underwent musculoskeletal ultrasound scans that revealed evidence of symmetrical synovitis [122].

However, there is still a lack of concrete evidence that the virus is present in the synovial fluid of the joints [123,124,125]. There are two theories regarding the relationship between COVID-19 and articular manifestations. One suggests that COVID-19 with viremia or a cytokine storm might cause viral arthritis, or it may be a non-specific consequence of the cytokine storm accompanying the symptomatic forms of the disease. However, no confirmed cases have been reported so far [125]. Another theory suggests that arthritis may be triggered by an inflammatory response to the systemic condition caused by COVID-19, leading to reactive arthritis [126].

Musculoskeletal ultrasounds allow for an earlier diagnosis and intervention for evolved diseases due to its high sensitivity for detecting articular changes, which is seven times more sensitive than plain radiography [122]. It is an easy and safe scanning tool for evaluating the articular changes. It can also assess the inflammation in joint diseases such as rheumatoid arthritis and osteoarthritis, even in the preliminary stages [127,128]. Additionally, it provides a real-time dynamic examination, offers physiological information, and is superior in distinguishing between fluid and solid materials [129].

The small number of results may be due to certain imaging services being prioritised during the pandemic due to their dominance in detecting COVID-19; other tests were either put on hold or had limited access as a precautionary measure against the viral infection [130,131]. At that time, the focus was on controlling and preventing the spread of the virus to avoid catastrophic outcomes for the healthcare system and increased mortality. Furthermore, the research scope was mainly aimed at disease prevention and avoiding critical illness in the case of infection, which is totally understandable; however, exploring the possible long-term effects of this disease is needed.

Individuals with chronic illnesses experience ongoing symptoms for over a month after a confirmed or suspected COVID-19 infection that cannot be explained by any other cause are considered to be cases of post-acute COVID syndrome (PACS) or long COVID. According to the Office of National Statistics (ONS), as of March 2023, almost 3% of the UK population has long COVID [132]. Research into this group is still lacking; the results after exposure to SARS-CoV-2 suggest that there is an imbalance in BTMs and a reduction in BMD. It is assumed that individuals with long COVID may have a greater risk due to their chronic condition. Potential causes of the condition may include the invasion of cells by SARS-CoV-2, inflammatory and immune responses, and the effects of the critical illness. These factors can lead to various symptoms and may result in abnormal changes in bones and joints [133]. Two papers systematically evaluated the literature on long COVID biomarkers and clarified that there is a significant elevation of acute phase markers such as CRP and cytokines/chemokines (such as TNF-α, INF-γ, and IL6) in long COVID patients [134,135]. Furthermore, one study demonstrated that patients with long COVID, assessed six months after hospital discharge, exhibited significantly lower levels of 25(OH) vitamin D, which was identified as an independent risk factor for the development of long COVID [136]. As mentioned previously, these factors may contribute to an imbalance in the bone remodelling process, resulting in a decreased bone volume.

## 5. Implication for Practice and Research

As we approach the five-year anniversary of the pandemic, many studies have primarily focused on the short-term effects of COVID-19 on bone and/or joint health. Short-term bone loss is anticipated in individuals on prolonged bed rest and those treated with corticosteroids, and is also commonly observed in severe cases of COVID-19. Prolonged immobility reduces mechanical loading on bones, leading to accelerated bone resorption and decreased bone formation, which has been well-documented in studies involving hospitalised or bed-bound patients. Additionally, corticosteroids, such as dexamethasone and prednisone, are frequently administered to manage severe COVID-19 symptoms; however, they are known to induce bone loss by impairing calcium absorption, increasing calcium excretion, and inhibiting osteoblast function. Both factors contribute to a heightened risk of osteoporosis and fractures, emphasising the need for careful management of bone health in these patients. Together, these factors significantly heighten the risk of osteoporosis and fractures, underscoring the need for careful management of bone health in these patients, as this is likely to impact their fracture risk.

This systematic review did not identify any studies meeting the inclusion criteria on the impact of long COVID-19 on bone and/or joint health, highlighting the need for research in this area. Such studies are crucial to advancing our understanding of long COVID-19′s musculoskeletal effects, which are expected to significantly influence clinical practice. Future clinical trials should also evaluate the effectiveness of interventions such as pharmacological treatments on bone and joint outcomes in long COVID-19 patients. These efforts will be essential in shaping clinical guidelines for managing these patients, including the development of early detection and screening tools. Ongoing studies, such as the ERASE-LC trial in the UK [137], may contribute to answering some of the unresolved questions in this field and addressing some of these important gaps in our knowledge.

Additionally, more longitudinal studies are warranted to assess the impact of COVID-19 on bone density and joint function across diverse populations and comorbidities. These studies should incorporate a range of imaging and laboratory methodologies to thoroughly investigate bone and joint health. Collaborative efforts utilising diagnostic tools such as DXA for bone density and ultrasound for joint assessments, along with relevant blood tests, could help elucidate the biological mechanisms linking COVID-19 to changes in bone and joint health, particularly inflammation and cytokine responses. Furthermore, advocating for dietary and lifestyle modifications such as increased vitamin D intake, alongside regular physical activity, is essential. Raising patient awareness about the potential long-term effects of COVID-19 on musculoskeletal health and promoting proactive management strategies are crucial steps in mitigating these impacts.

## 6. Limitations

It is important to note that this study has several limitations. First, even after more than three years since the start of the COVID-19 pandemic, there is still a lack of related articles on many aspects of bone health services, limiting our knowledge. Second, due to the inclusion of only English-language papers, a language bias is anticipated. Third, existing studies sometimes report discrepant materials, making it difficult to discuss the desired details. This is due to the fact that our understanding of the pandemic and its effects is rapidly evolving, and the results of studies were heterogeneous and come from different communities with different social and indigenous situations. Factors such as age, gender, ethnicity, comorbidities, alcohol, smoking, nutrition, etc., are also important when considering bone health. Fourth, because of their intrinsic vulnerability to bias and confounding, observational trials are limited in their capacity to establish causality. Their advantages, however, lie in the fact that, in comparison to randomised controlled trials (RCTs), they more closely mimic routine clinical practice in terms of the medical interventions and diverse patient populations they involve [138]. Lastly, the present study was intended to be a meta-analysis. Due to factors such as the scarcity and heterogeneity of existing articles with regard to the effect size measures and outcomes as well as co-morbidities, it was not possible to draw conclusions.

## 7. Conclusions

This is the first and most comprehensive systematic review of studies that investigated the effect of the SARS-CoV-2 virus on BMD, BTMs, and joints. We found that COVID-19 has a negative impact on bone health and can lead to increased bone regulatory markers and decreased bone formation and absorption, and BMD. There is a complex relationship between BTMs and inflammation due to COVID-19, which may disrupt the normal bone regulatory mechanism. Currently, there are no studies evaluating the association between long COVID and bone and joint health. Therefore, it would be beneficial to measure BMD using DXA and monitor BTMs (CTX and P1NP) and joints using ultrasound. Hence, more research with a focus on musculoskeletal comorbidities associated with COVID-19 and long COVID is needed. One key finding from this review is that elderly and frail individuals, as well as those who are already osteopenic or osteoporotic before contracting COVID-19, may be at a higher risk. Thus, it is vital for these individuals to be monitored for bone loss.

## Figures and Tables

**Table 1:** Keywords and algorithm developed for database search.

Keywords	
“SARS-CoV-2”	OR
“COVID 19”	
“Long COVID”	
“long-haulers COVID-19”	
“post-acute COVID-19”	
“post-COVID syndrome”	
“post-acute COVID-19 syndrome”	
“post-acute COVID-19 sequelae”	
“Persistent COVID-19 symptoms”	
“Prolonged COVID-19”	
“post-COVID-19 manifestations”	
“Post-acute sequelae of COVID-19”	
“PASC”	
“Chronic COVID syndrome”	
AND	
“Bone Health”	OR
“Bone Biomarkers”	
“P1NP”	
“CTX”	
“BSAP”	
“Bone Health Biomarkers”	
“Bone Turnover Markers”	
“Bone Density”	
“Bone Mineral Density”	
“Bone Densitometry”	
“DEXA”	
“DXA”	
“Ultrasound”	
“Ultrasonography”	
“Musculoskeletal Ultrasound”	
“MSK Ultrasound”	
“Joints Ultrasound”	
“Articular Ultrasound”	
“Hand Ultrasound”	
“Knee Ultrasound”	

PASC: post-acute sequelae of COVID-19; P1NP: pro-collagen type I N-terminal propeptide; CTX: C-terminal telopeptide of type 1 collagen; BSAP: bone-specific alkaline phosphatase; DEXA/DXA: dual-energy X-ray absorptiometry.

**Table 2:** Information for the studies.

Author, Year, Country	Study Design	Aim	Participation (Case/Control)	Age Mean (SD)	Male (%)	Comorbidities	Assessment	Hospitalisation	COVID-19 Status at Assessment	Follow-Up
(Kerschan-Schindl et al.,2023) Austria [36]	Case–Control	Evaluate the BTMs in COVID-19 patients requiring hospitalisation	50 (25/25)	Median C: (67 [52; 81]) NC: (66 [53; 80.5])	C: 44 NC: 44	N/A	BTMs	Yes	Acute	-
(Elmedany et al., 2022) Saudi Arabia [37]	Single-Centre Retrospective	Study the impact of COVID-19 infection on BMD in osteoporotic and osteopenic patients	100 (56/44)	C: 62.84 ± 9.05 NC: 61.64 ± 6.10	C: 26.8 NC: 25	patients diagnosed with osteoporosis or osteopenia	DXA	N/A	3 mo. post-infection	9 mo. and 1 year
(Berktas et al., 2022) Turkey [38]	Single-Centre Retrospective	Evaluate the impact of COVID-19 illness and treatment on the bone health of surviving patients	58 (58/N/A)	C: 63.2 ± 9.6	C: 69	N/A	CT	Yes	Acute	mean of 81 days (±48)
(Moga et al., 2022) Romania [39]	Prospective Cross-Sectional	Compare the presence of sarcopenia and osteoporosis in patients with recent COVID-19 infection to that of the general population	157 (86/71)	C: 55.67 ± 4.43 65.45 ± 2.66 75.96 ± 3.26 NC: 55.50 ± 4.10 65.46 ± 33.92 76.15 ± 3.34	C: 45.35 NC: 42.26	patients with various musculoskeletal disorders (neurological or degenerative)	(DXA)	Yes	1 mo. post-infection	-
(Al-Azzawi et al., 2022) Iraq [40]	Case–Control	Determine if there is a disruption of bone homeostasis balance in Iraqi post-COVID-19 patients	130 (80/50)	Women: 18–45 Men: 18–60 Median W: ([18; 45] 31) M: ([18; 60] 39)	N/A	N/A	(DXA and BTMs)	No	3 mo. post-infection	-

BTMs: bone turnover markers; BMD: bone mineral density, CT: computed tomography; DXA: dual-energy X-ray absorptiometry; C: COVID-19; NC: non-COVID-19 control.

**Table 3:** BMD results based on time of measurement.

		Bone Mineral Density Changes												
Study	Body Site	Modality	Acute	After 1 Month		After 3 Months			After 9 Months			After 21 Months		
(Elmedany et al., 2022) [37] Mean ± SD g/cm^2^	Femur and lumbar vertebra	DXA	-	-		Lumbar vertebra	C	0.96 ± 0.11	Lumbar vertebra	C	0.91 ± 0.11	Lumbar vertebra	C	0.96 ± 0.11
							NC	0.92 ± 0.11		NC	0.92 ± 0.10		NC	0.96 ± 0.11
						Femur	C	0.88 ± 0.12	Femur	C	0.84 ± 0.11	Femur	C	0.89 ± 0.13
							NC	0.89 ± 0.11		NC	0.88 ± 0.13		NC	0.89 ± 0.13
(Berktas et al., 2022) [38] Mean ± SD mg/cm^3^	T11, T12, and L1 vertebrae	CT	119.2 ± 36.8	-		110.1 ± 38.5			-			-		
(Moga et al., 2022) [39] T score ± SD g/cm^2^	Femur and lumbar vertebra	DXA	-	C	−3.55 ± 0.72				-			-		
				NC	−2.09 ± 1.06									
(Al-Azzawi et al., 2022) [40] T score % Mean ± SD g/cm^2^	Not specified	DXA	-	-		C		−0.43 ± 0.94	-			-		
						NC		0.45 ± 0.64						

C: COVID-19; NC: non-COVID-19 control; CT: computed tomography; DXA: dual-energy X-ray absorptiometry; T: thoracic spine; L: lumbar spine; and SD: standard deviation.

**Table 4:** Bone turnover marker results based on COVID-19 phase.

Study	COVID Phase	Bone Turnover Markers				
(Kerschan-Schindl et al.,2023) [36] Median [quartiles]	Acute	Biomarker		COVID (n = 25)	Control (n = 25)	*p* Value
		Resorption	CTX-1 [ng/mL]	0.172 [0.097; 0.375]	0.462 [0.300; 0.649]	0.011
			TRAP [U/L]	2.782 [2.129; 3.505]	3.335 [2.535; 4.224]	0.115
		Formation	OC [ng/mL]	10.50 [6.49; 16.26]	15.33 [11.85; 19.63]	0.025
			BAP [µg/L]	14.98 [10.67; 17.81]	14.98 [12.08; 18.96]	0.840
		Regulatory	SOST [pmol/L]	37.52 [25.30; 60.11]	27.98 [24.05; 39.24]	0.025
			Dkk1 [pmol/L] (n = 24)	23.16 [19.77; 34.73]	21.31 [15.04; 24.69	0.026
			OPG [pmol/L]	4.94 [3.39; 7.30]	5.07 [3.15; 6.62]	0.638
(Al-Azzawi et al., 2022) [40] Mean ± SD	After 3 months	Biomarker		COVID (n = 80)	Control (n = 50)	*p* Value
		Regulatory	OPG (ng/mL)	2.24 ± 1.0	0.7 ± 0.21	0.001

CTX-1: cross-linked C-telopeptide of type I collagen; TRAP: tartrate-resistant acid phosphatase; OC: osteocalcin; BAP: bone-specific alkaline phosphatase; SOST: sclerostin; Dkk1: dickkopf-1; OPG: osteoprotegerin.

**Table 5:** Newcastle–Ottawa Scale scores for each paper.

Study	Selection	Comparability	Outcome/Exposure	Overall Score
(Kerschan-Schindl et al., 2023) [36]	**	*	***	6 stars [Low Risk]
(Elmedany et al., 2022) [37]	****	*	***	8 stars [Low Risk]
(Berktas et al., 2022) [38]	*		**	3 stars [High Risk]
‡ (Moga et al., 2022) [39]	****	*	***	8 stars [Low Risk]
(Al-Azzawi et al., 2022) [40]	**		***	5 stars [Medium Risk]

Newcastle-Ottawa Scale score provides a rating system using stars. For cohort and case–control studies, a risk of bias overall 6 stars or above: low risk; 4 to 5 stars: medium risk; and 1 to 3 stars: high risk. For the cross-sectional study ‡, very good studies: 9–10 points; good studies: 7–8 points, satisfactory studies: 5–6 points; unsatisfactory studies: 0 to 4 points.
